# Automated 3D chest CT muscle segmentation–derived model development and explanation in discrimination of normal, preserved ratio impaired spirometry, and COPD: a multicenter study

**DOI:** 10.1186/s13244-026-02384-4

**Published:** 2026-08-27

**Authors:** Yi Wang, Weihao Zhai, Qian Zhou, Xiuxiu Zhou, Yueze Li, Taohu Zhou, Xin’ang Jiang, Qianxi Jin, Yanming Ge, Peng Dong, Ruoyao Wang, Shiyuan Liu, Li Fan

**Affiliations:** 1 Department of Radiology, Second Affiliated Hospital of Naval Medical University, Shanghai, China; 2 School of Medical Imaging, Shandong Second Medical University, Weifang, China; 3 College of Health Sciences and Engineering, University of Shanghai for Science and Technology, Shanghai, China; 4 Department of Nuclear Medicine, Huadong Hospital Affiliated to Fudan University, Shanghai, China

**Keywords:** COPD, CT, Muscle, PRISm, Segmentation

## Abstract

**Objectives:**

To develop and validate an automated 3D chest CT muscle segmentation model using an exploratory normalization method to discriminate normal, preserved ratio impaired spirometry (PRISm), and chronic obstructive pulmonary disease (COPD) subjects.

**Materials and methods:**

In this retrospective study, we used TotalSegmentator to acquire 3D pectoral muscle (PM) and erector spinae muscle (ESM) volumes from chest CT, normalizing them to height and vertebral bone volume, respectively. 2D quantitative parameters were acquired and normalized. The support vector machine models were developed based on normalized 3D and 2D muscle parameters, respectively, with ten-fold cross-validation. Receiver operating characteristic curves were used to analyze the discriminative performance of models, and the SHapley Additive exPlanations algorithm was used to quantify the importance of each parameter.

**Results:**

1660 subjects (616 normal, 646 PRISm, 398 COPD) (median age, 66 years [IQR: 60–72 years]; 1014 males) were included and divided into training set (*n* = 1162), test set (*n* = 498). In the test set, the 3D model (macro-average AUC, 0.735; micro-average AUC, 0.733) outperformed the 2D model (macro-average AUC, 0.646; micro-average AUC, 0.665), and 3D ESM density was the most relevant muscle parameter. 3D muscle density showed a decreasing trend from normal to PRISm to COPD. In COPD, females exhibited decreased density (difference range: −14.48 to −7.79; *p* < 0.001), while males had a more significant decrease in volume, especially pectoralis major volume index (difference: −11.18; 95% CI −15.57, −5.08; *p* < 0.001).

**Conclusions:**

The 3D muscle parameter model demonstrated higher discriminative performance than the 2D model for discriminating normal, PRISm, and COPD subjects.

**Key Points:**

- **Question** The discriminative performance of 3D CT-based muscle quantification in subjects with impaired pulmonary function remains unclear.
- **Findings** 3D muscle analysis achieved superior discrimination of normal, PRISm, and COPD subjects, with density-related parameters showing consistent group differences, particularly ESM density.
- **Critical relevance** By comparison with conventional 2D assessment, 3D CT-based muscle quantification showed better discrimination of normal, PRISm, and COPD, which might advance the personalized precision and comprehensive assessment, including muscle alteration for subjects with impaired pulmonary function.

**Graphical Abstract:**

**Figure:**
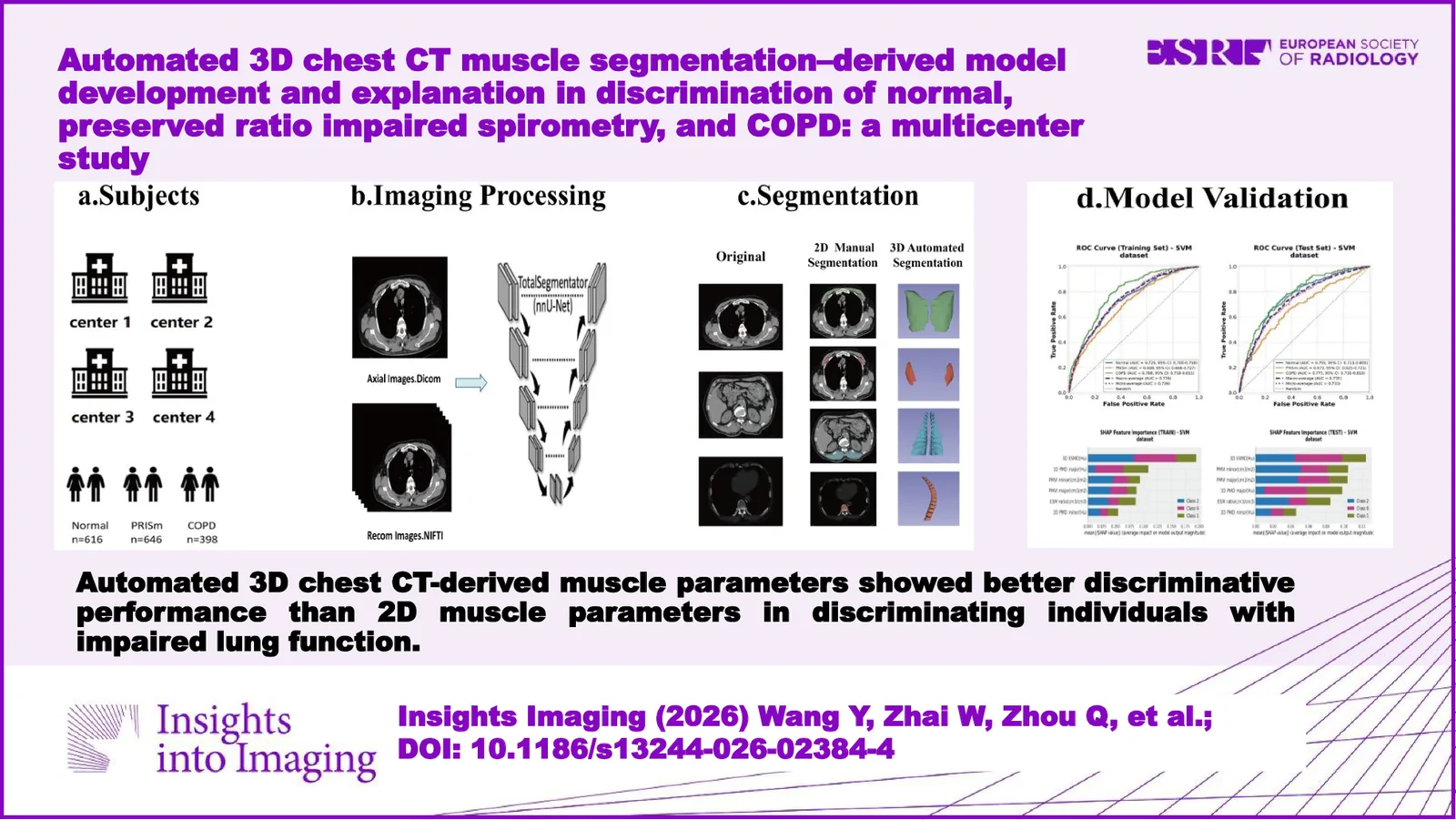

## Introduction

Chronic obstructive pulmonary disease (COPD) presents a major global public health burden due to its widespread occurrence, substantial disability, and excess mortality [1–3]. It manifests as chronic, irreversible airflow obstruction and is frequently accompanied by systemic comorbidities—including sarcopenia, cardiovascular disease, and osteoporosis [4]. Sarcopenia is highly prevalent in COPD patients and has significant negative impacts on pulmonary function, exercise capacity, and quality of life. Although airflow restriction is a criterion for diagnosing COPD, there is substantial evidence that forced expiratory volume in 1 s (FEV₁) is weakly associated with mortality risk or health-related quality of life [5, 6]. However, skeletal muscle is now recognized as a more reliable predictor of clinical outcomes and hospitalization risk than traditional measures of lung function [7–9].

The overall changes in muscle in COPD patients can be directly measured through imaging techniques. Usually, the segmentation of the pectoral muscle (PM) is typically performed at the level of the aortic arch [10], with quantitative analysis of the cross-sectional area (CSA) and average density at this level. Multiple studies based on this approach have confirmed that both the PM cross-sectional area (PM_CSA_) and density in COPD patients are significantly lower than those in normal control subjects [11–13]. The erector spinae muscle (ESM) has also been reported to be correlated with fat-free mass, symptoms, disease severity, and prognosis in patients with lung disease [14, 15]. While these measurements were all based on the 2D images, which could not reflect the whole changes accurately. Therefore, 3D segmentation and measurement may provide a more comprehensive assessment of muscle alterations. Recent advances in deep learning have facilitated the development of automated 3D segmentation algorithms, enabling efficient delineation of anatomical structures on CT images and more accurate estimation of muscle volume [16–18]. This enables diverse clinical applications, including diagnosis, treatment planning, and disease monitoring in personalized healthcare.

Muscle parameters derived from 3D segmentation have not been widely compared among normal, Preserved Ratio Impaired Spirometry (PRISm), and COPD groups. PRISm, a common but understudied spirometric pattern [19], represents a heterogeneous state linked to cardiopulmonary comorbidities and increased risk of progression [20–22]. Recently, research on PRISm has expanded rapidly, focusing on its epidemiology and clinical subtypes. However, few studies have included PRISm in the evaluation of muscle changes. Additionally, the scanning range of chest CT is difficult to cover the entire ESM, and a standardized evaluation method suitable for this muscle has not yet been established.

Therefore, we aim to develop and validate a model, then conduct a comparative analysis based on PM and ESM muscle parameters among three groups with automated 3D chest CT segmentation by using an exploratory normalization strategy, and further investigate whether this method outperforms conventional 2D segmentation analysis.

## Materials and methods

### Patients

**Fig. 1:**
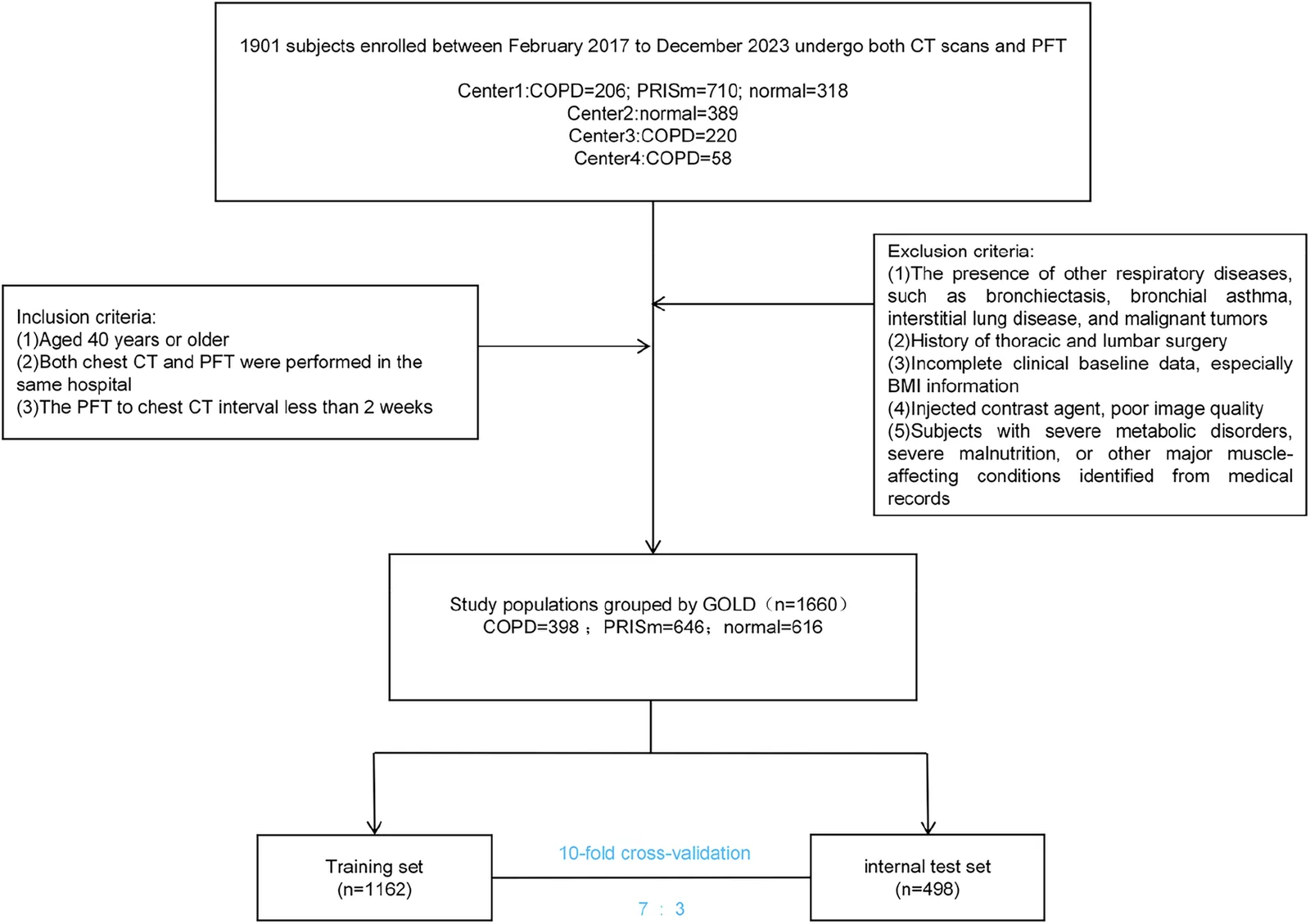
The flowchart for study subjects

**Fig. 2:**
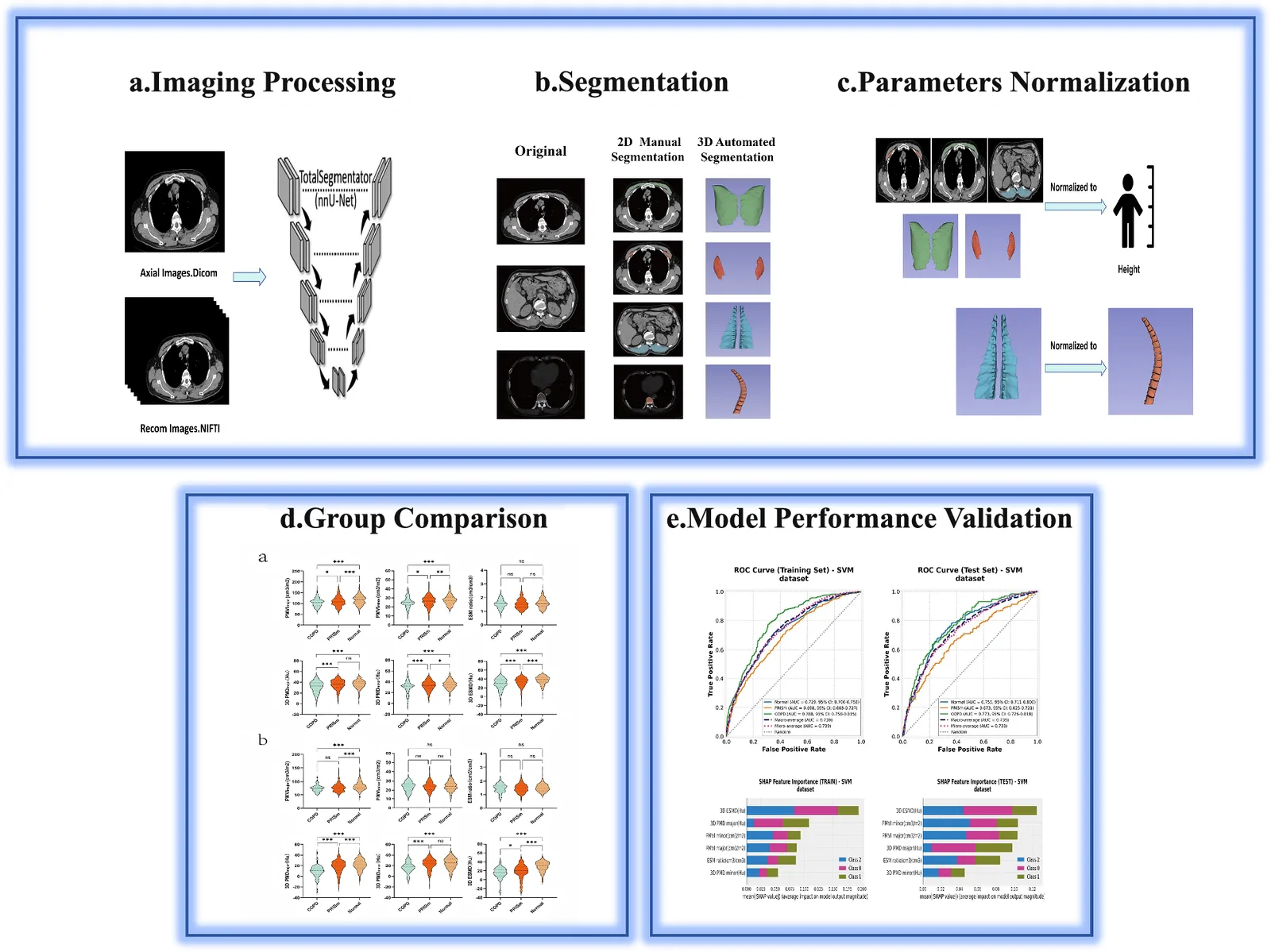
The flowchart of the study workflow. **a** Imaging processing: chest CT DICOM images from normal, PRISm, and COPD were collected. Axial images were selected and converted to NIFTI format for 3D segmentation using TotalSegmentator and for 2D segmentation using 3D Slicer. **b** Segmentation: 2D and 3D segmentation of pectoralis major (PM_major_), pectoralis minor (PM_minor_), erector spinae muscle (ESM), and vertebrae at the same level as ESM. **c** Parameters normalization: PM_major_, PM_minor_ (2D/3D), and ESM (2D) were normalized by dividing by the square of body height, whereas ESM (3D) was normalized by the volume of the vertebrae at the same level. **d** Group comparison: overall comparison of muscle parameters among the three groups, stratified by gender. **e** Model performance validation: ROC curves were used to evaluate model performance, and the importance of each parameter was assessed based on SHAP values

This retrospective study enrolled 1901 subjects from the CSD-COPD cohort (CT Standardized Data for Chronic Obstructive Pulmonary Disease; ChiCTR2300069929), including normal, PRISm, and COPD subjects who underwent chest CT and pulmonary function tests for clinical practice between February 2017 and December 2023 at four centers, including The Second Affiliated Hospital of Naval Medical University (Center 1), Tongji Hospital, Tongji University School of Medicine (Center 2), The Affiliated Hospital of Shandong Second Medical University (Center 3), and Weifang Second People’s Hospital (Center 4). The inclusion criteria were as follows: (1) aged 40 years or older; (2) both chest CT and PFT were performed in the same hospital; (3) the interval between PFT and chest CT was less than 2 weeks. The exclusion criteria were as follows: (1) the presence of other respiratory diseases, such as bronchiectasis, bronchial asthma, and malignant tumors; (2) history of thoracic and spinal surgery; (3) incomplete clinical baseline data, especially BMI information; (4) injected contrast agent, poor image quality; (5) subjects with severe metabolic disorders, severe malnutrition, or other major muscle-affecting conditions identified from medical records. Subjects’ inclusion criteria and study workflow were available (Figs. 1 and 2). This study was approved by the institutional review boards at 4 centers, and informed consent was waived due to the retrospective nature of this study (2022SL068).

### Clinical features and pulmonary function test

The clinical information, including age, sex, height, weight, and lung function results, was obtained from the electronic medical record.

Pulmonary function parameters (FEV_1_/FVC%, FEV_1_% predicted) were measured with a PFT apparatus (CHEST Multifunction Spirometer HI-801, Japan; Carefusion GmbH; Masterscreen PFT Pro). Subjects were grouped according to the Global Initiative for Chronic Obstructive Lung Disease Chronic Obstructive Lung Disease (GOLD) [23]: COPD, FEV_1_/forced vital capacity (FVC) < 0.7, with an increase of FEV_1_ < 200 mL after the use of a bronchodilator; PRISm, FEV_1_/FVC ≥ 0.7 and the FEV_1_% predicted < 80%; normal, FEV_1_/FVC ≥ 0.7 and the FEV_1_% predicted ≥ 80%.

### CT examinations and muscle composition analysis

All examinations were conducted using baseline CT scans, and the specifications of the CT scanners employed are listed in (Table S1). The subjects were trained to breathe hold on deep inspiration for a full lung scan.

Both 3D and 2D muscle composition analyses were performed as follows:

### 3D muscle composition analysis

**Fig. 3:**
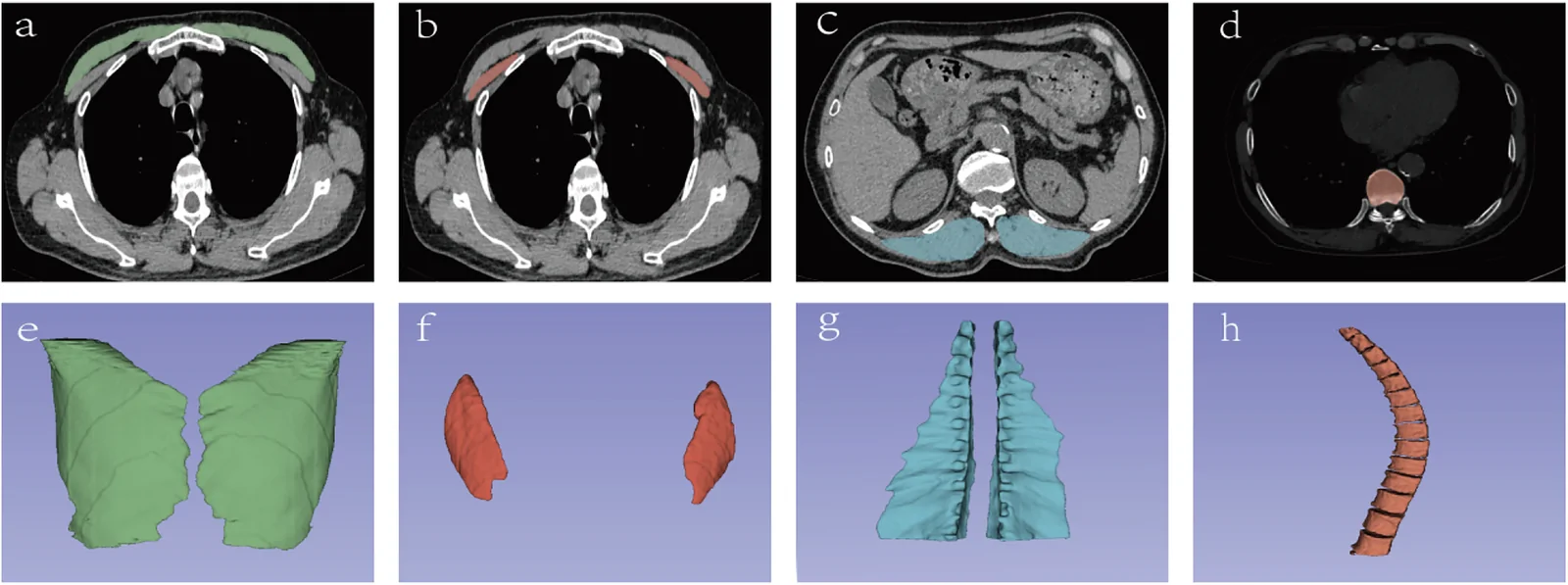
Representative CT images for the measurement of cross-sectional area (CSA) and volume of the pectoralis major (PM_major_), pectoralis minor (PM_minor_), erector spinae (ESM) muscles and vertebral. **a**, **b** The CT axial slice of the PM_CSA_ (bilateral PM_major_ are shaded in green and PM_minor_ are shaded in red). **c** The CT axial slice of the ESM_CSA_ (bilateral ESM are shaded in blue). **d** The vertebral body CSA (orange-shaded area). **e**–**h** PM_major_, PM_minor_, ESM, and vertebral body volume (within the field of view of the chest CT scan)

TotalSegmentator is an open-access tool for whole-body CT segmentation suitable for clinical and research applications [24]. It was used for automatic segmentation of pectoralis major (PM_major_) (Fig. 3e), pectoralis minor (PM_minor_) (Fig. 3f), ESM (Fig. 3g), and volume of the vertebral bone corresponding to the ESM (Fig. 3h), followed by visual inspection and manual correction when necessary. Subsequently, 150 CT segmentation results (50 normal, 50 PRISm, and 50 COPD) were randomly selected. Two blinded radiologists (T.H.Z. and X.X.Z.) independently reviewed and corrected the segmentations using 3D Slicer (version 5.6.2), and interobserver agreement of the derived muscle parameters was assessed using the intraclass correlation coefficient (ICC). In order to evaluate the body composition accurately, we perform the standardization process. For PM_major_ and PM_minor_, the chest CT scanning range can cover both of them. We used muscle volume index (MVI) (volume divided by height squared, in units of cm^3^/m^2^) to evaluate the PM muscle volume, such as PM_major_ volume index (PMVI_major_), PM_minor_ volume index (PMVI_minor_). Due to the limited chest CT scanning range, the ESM cannot be fully covered. We therefore defined the ESM ratio (ESM volume divided by corresponding vertebral body bone volume, in units of cm^3^/cm^3^). Although a standardized method for ESM normalization is lacking, a previous study has used bone volume as a reference [25]. Given its close anatomical relationship with the ESM and its relatively stable structural characteristics compared with bone mineral density [26], we adopted vertebral bone volume for normalization. Mean CT attenuation values, expressed in Hounsfield units (HU), were used to assess muscle density of the PM and ESM, including PM_major_ density (PMD_major_), PM_minor_ density (PMD_minor_), and ESM density (ESMD) [27].

### 2D muscle composition analysis

We performed a quantitative assessment of PM and ESM using 3D Slicer at the level of the aortic arch and the lower edge of the 12th thoracic vertebra. On the first axial slice above the aortic arch, the left and right PMs_major_, PMs_minor_ were manually segmented from voxels within −50 to +90 (HU) (Fig. 3a, b) [28]. At the same CT threshold, on a single axial slice at the lower margin of the 12th thoracic vertebra, the left and right ESMs were similarly outlined [15] (Fig. 3c). The sum of the bilateral muscle areas was adopted as the PM_CSA_ (major/minor) and ESM cross-sectional area (ESM_CSA_). This work was also performed by the same two radiologists. We used the skeletal muscle index (SMI) (CSA divided by height squared, in units of cm^2^/m^2^) to standardize muscle CSA [29], such as PM_major_ index (PMI_major_), PM_minor_ index (PMI_minor_), and ESM index(ESMI). For the assessment of PM_CSA_ and ESM_CSA_ density, we employed the 2D CT mean attenuation (in units of HU).

Considering the inevitable physiological differences between male and female muscles, we stratified subjects by gender [30]. Moreover, we also conducted the subgroup analyses by age (< 65 years vs ≥ 65 years) and BMI (< 25 kg/m^2^ vs ≥ 25 kg/m^2^).

### Model development, validation, and explanation

We developed support vector machine (SVM) classification models mainly based on two groups: 3D parameters (muscle volume index, density, and ESM ratio), and 2D parameters (muscle index and density). The data was randomly split into training and test sets in a 7:3 ratio, performing 10-fold cross-validation. SVM model used an RBF kernel with fixed hyperparameters (C = 10, gamma = ‘scale’, class_ weight = ‘balanced’). To evaluate 3D model robustness, we performed subgroup analyses by gender. The one-vs-rest AUCs, macro-average AUCs, and micro-average AUCs of different models were calculated for different models.

To assess the contribution of muscle parameters to SVM classification decisions, we used SHapley Additive exPlanations (SHAP) as a post hoc interpretability method and ranked features by mean absolute SHAP values. SHAP analysis was performed using the Kernel Explainer package (version 0.40.0) in Python (version 3.8.0).

### Statistical analysis

In this study, statistical analyses were performed by R software (version 4.1.0), GraphPad Prism (version 10.2.0, GraphPad Software), and IBM SPSS Statistics (version 26.0; IBM Corp). The associations between pulmonary function and 3D muscle parameters were examined using partial correlation analysis with gender as a covariate. Differences between the three groups for continuous variables were determined using ANOVA or Kruskal–Wallis, depending on the data type. Then the *p*-values were adjusted using the Benjamini–Hochberg method. Receiver operating characteristic curves and corresponding AUCs were compared using the DeLong test. All statistical tests were two-sided, and a *p* < 0.05 was considered to indicate strength of evidence against the null hypothesis.

## Results

### Clinical characteristics

A total of 1660 consecutive subjects (1014 males, 646 females; median age, 66 years) were included. Among these subjects, 616 (37.1%) were normal, 646 (38.9%) were PRISm, and 398 (24.0%) had COPD. The COPD group comprised 17 (4.30%) subjects with GOLD 1, 165 (41.41%) with GOLD 2, 157 (39.57%) with GOLD 3, and 59 (14.72%) with GOLD 4. Significant differences were observed in age, gender, BMI, and most muscle parameters (3D/2D) among the three groups (Table 1). In addition, most muscle parameters (3D/2D) demonstrated weak correlations with pulmonary function (Table S2).

**Table 1:** Participant characteristics at baseline

Variable	COPD (*n* = 398)	PRISm (*n* = 646)	Normal (*n* = 616)	*p*-value
Age, years	69.39 (65.00, 74.00)	66.00 (60.00, 72.00)	63.00 (56.00, 70.00)	< 0.001
Male, %	321 (80.7)	438 (67.8)	255 (41.4)	
BMI, kg/m^2^	23.04 (21.45, 25.14)	25.64 (23.18, 28.21)	24.27 (22.23, 26.67)	< 0.001
Smoking status				< 0.001
Never-smoker, %	140 (35.2)	426 (65.9)	514 (83.4)	
Former-smoker, %	154 (38.7)	80 (12.4)	31 (5.1)	
Current-smoker, %	104 (26.1)	140 (21.7)	71 (11.5)	
GOLD stage, %				
GOLD I	4.30	-	-	
GOLD II	41.41	-	-	
GOLD III	39.57	-	-	
GOLD IV	14.72	-	-	
PMVI_major_, cm³/m²	100.76 (82.45, 108.92)	98.56 (79.96, 116.53)	93.64 (80.17, 115.71)	0.396
PMVI_minor_, cm³/m²	24.78 (21.26, 27.86)	25.54 (21.63, 29.21)	25.75 (22.12, 29.04)	< 0.05
ESM ratio, cm³/cm³	1.53 ± 0.33	1.51 ± 0.34	1.54 ± 0.31	0.215
3D PMD_major_, HU	30.95 (17.79, 36.12)	31.87 (22.05, 39.76)	30.29 (21.37, 36.95)	< 0.01
3D PMD_minor_, HU	29.99 (19.19, 34.42)	30.65 (23.56, 37.06)	30.18 (22.20, 36.41)	0.053
3D ESMD, HU	27.39 (17.60, 36.70)	32.09 (24.03, 38.80)	34.60 (26.35, 40.56)	< 0.001
PMI_major_, cm²/m²	7.04 (5.72, 8.11)	8.14 (6.53, 10.27)	7.92 (6.63, 9.57)	< 0.001
PMI_minor_, cm²/m²	2.51 (2.18, 3.08)	2.85 (2.36, 3.33)	2.72 (2.26, 3.34)	< 0.001
ESMI, cm²/m²	10.31 ± 2.34	11.70 ± 2.68	12.27 ± 2.53	< 0.001
2D PMD_major_, HU	35.57 (24.54, 47.20)	38.20 (29.57, 45.64)	37.67 (29.99, 44.00)	0.121
2D PMD_minor_, HU	36.72 (27.07, 42.17)	38.57 (32.15, 44.50)	38.61 (32.85, 45.24)	< 0.001
2D ESMD, HU	35.33 (28.37, 43.24)	38.96 (32.64, 43.99)	39.55 (32.85, 44.91)	< 0.001

*PMVI*_*major*_ pectoralis major volume index, *PMVI*_*minor*_ pectoralis minor volume index, *ESM ratio* erector spinae muscle volume/the corresponding vertebral bone volume, *PMI*_*major*_ pectoralis major index, *PMI*_*minor*_ pectoralis minor index, *ESMI* erector spinae muscle index, *PMD*_*major*_ pectoralis major density, *PMD*_*minor*_ pectoralis minor density, *ESMD* erector spinae muscle density

### Interobserver reliability assessment

After manual correction, ICCs and 95% CIs for muscle parameters were as follows: PM_major_ volume (ICC = 0.990, 95% CI: 0.988–0.993); PMD_major_ (ICC = 0.987, 95% CI: 0.985–0.991); PM_minor_ volume (ICC = 0.988, 95% CI: 0.986–0.992); PMD_minor_ (ICC = 0.998, 95% CI: 0.996–0.999); ESM volume (ICC = 0.996, 95% CI: 0.993–0.998); ESMD (ICC = 0.999, 95% CI: 0.997–0.999), indicating high reliability.

### Comparison of 3D muscle parameters across normal, PRISm, and COPD subjects

**Fig. 4:**
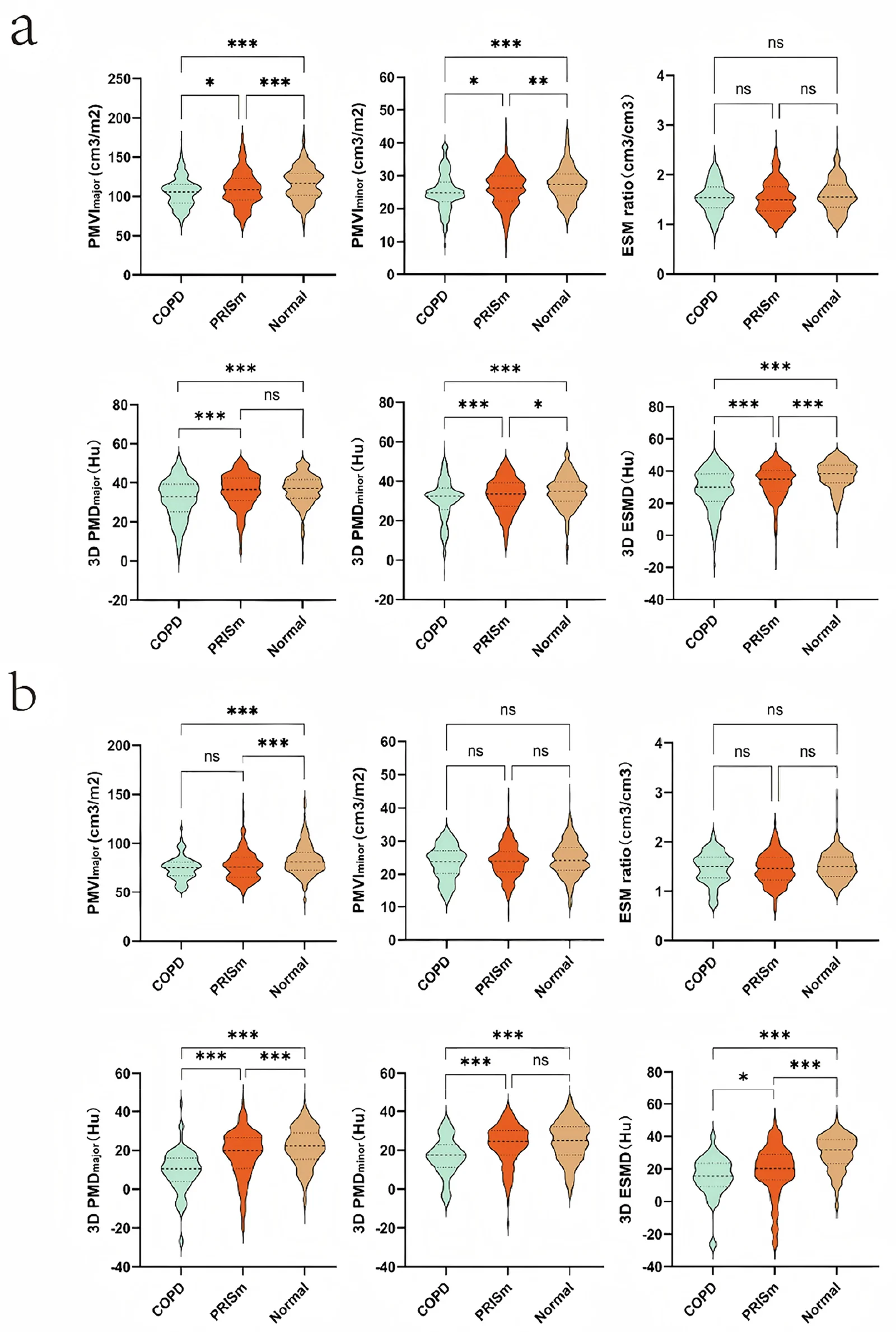
Distributions of the muscle volume index (cm^3^/m^2^), density (HU), and ESM ratio (cm^3^/cm^3^) of the PM_major_, PM_minor_ and ESM across normal, PRISm, and COPD (**a** male; **b** female)

Overall, muscle density parameters showed a clearer gradient from normal to PRISm to COPD than volume-related parameters (Fig. 4).

#### Comparison between COPD and normal

PMVI_major_ (male/female), PMVI_minor_ (male), and density parameters (male/female) were lower in the COPD group than in the normal group (all *p* < 0.01). Moreover, density parameters were significantly lower in the female COPD group than in the normal group (male difference range: −11.18 to −2.3; female difference range: −14.48 to −7.79; all *p* < 0.001). Males showed greater between-group differences in PMVI than females, especially for PMVI_major_ (male difference: −11.18; 95% CI −15.57, −5.08; female difference: −6.10; 95% CI −11.11, −4.28; all *p* < 0.001) (Table 2).

**Table 2:** Comparison of the 3D muscle parameters stratified by gender

Variables	Male		Female	
	Difference (95% CI)	Adjusted *p*-value	Difference (95% CI)	Adjusted *p*-value
PMVI_major_ (cm^3^/m^2^)		< 0.001		< 0.001
COPD vs PRISm	−2.97 (−5.97, 1.32)	< 0.05	−1.53 (−6.99, 0.90)	0.101
COPD vs Normal	−11.18 (−15.57, −5.08)	< 0.001	−6.10 (−11.11, −4.28)	< 0.001
Normal vs PRISm	8.21 (3.07, 13.23)	< 0.001	4.57 (1.25, 7.41)	< 0.001
PMVI_minor_ (cm^3^/m^2^)		< 0.001		0.201
COPD vs PRISm	−0.91 (−1.76, −0.06)	< 0.01		
COPD vs Normal	−2.30 (−3.22, −1.38)	< 0.001		
Normal vs PRISm	1.39 (0.55, 2.23)	< 0.05		
ESM ratio (cm^3^/cm^3^)		0.097		0.193
COPD vs PRISm				
COPD vs Normal				
Normal vs PRISm				
PMD_major_ (HU)		< 0.001		< 0.001
COPD vs PRISm	−4.03 (−5.44, −2.63)	< 0.001	−9.75 (−11.90, −7.46)	< 0.001
COPD vs Normal	−5.23 (−6.67, −3.80)	< 0.001	−11.78 (−14.03, −9.76)	< 0.001
Normal vs PRISm	1.20 (−0.08, 2.48)	0.283	2.04 (0.43, 4.40)	< 0.01
PMD_minor_ (HU)		< 0.001		< 0.001
COPD vs PRISm	−2.61 (−4.04, −1.19)	< 0.01	−6.93 (−8.91, −5.46)	< 0.001
COPD vs Normal	−4.47 (−6.02, −2.92)	< 0.001	−7.79 (−10.20, −6.28)	< 0.001
Normal vs PRISm	1.86 (0.54, 3.18)	< 0.05	0.86 (−1.44, 3.22)	0.092
ESMD (HU)		< 0.001		< 0.001
COPD vs PRISm	−4.66 (−6.40, −2.92)	< 0.001	−9.34 (−13.41, −5.25)	< 0.001
COPD vs Normal	−8.59 (−10.40, −6.77)	< 0.001	−14.48 (−17.88, −11.09)	< 0.001
Normal vs PRISm	3.93 (2.44, 5.42)	< 0.001	5.14 (1.96, 7.90)	< 0.001

*PMVI*_*major*_ pectoralis major volume index, *PMVI*_*minor*_ pectoralis minor volume index, *ESM ratio* erector spinae muscle volume/the corresponding vertebral bone volume, *PMD*_*major*_ pectoralis major density, *PMD*_*minor*_ pectoralis minor density, *ESMD* erector spinae muscle density

#### The characteristics of PRISm in comparison with normal and COPD

The ESM density (male/female), PMVI (male), showed a decreasing trend across normal, PRISm, and COPD groups (all *p* < 0.05). The female PMVI_major_ in the normal group was also higher than in the PRISm group (*p* < 0.001). In addition, compared to the COPD group, other density parameters in the PRISm group were higher (all *p* < 0.01) (Table 2).

#### *Comparison of 3D muscle parameters across normal, PRISm, and COPD subjects stratified by BMI*

In BMI-stratified analysis (Tables 3 and 4), PMVI_major_ (male/female), PMVI_minor_ (male) were lower in COPD than in the normal group (all *p* < 0.01), and most density parameters in COPD were lower than normal and PRISm group (all *p* < 0.05).

**Table 3:** Comparison of 3D muscle parameters in male stratified by BMI

Variables	Male, BMI ≥ 25		Male, BMI < 25	
	Difference (95% CI)	Adjusted *p*-value	Difference (95% CI)	Adjusted *p*-value
PMVI_major_ (cm^3^/m^2^)		< 0.001		< 0.001
COPD vs PRISm	−1.42 (−6.39, 3.55)	0.877	−0.39 (−4.24, 3.47)	0.604
COPD vs Normal	−8.74 (−14.32, −3.16)	< 0.01	−9.33 (−13.37, −5.28)	< 0.001
Normal vs PRISm	7.32 (2.42, 12.22)	< 0.001	8.94 (4.28, 13.59)	< 0.001
PMVI_minor_ (cm^3^/m^2^)		< 0.01		< 0.001
COPD vs PRISm	−0.59 (−2.20, 1.13)	0.182	−0.02 (−1.80, 1.37)	0.289
COPD vs Normal	−1.21 (−2.97, 0.71)	< 0.05	−1.70 (−3.03, 0.02)	< 0.01
Normal vs PRISm	0.63 (−0.32, 1.95)	0.118	1.68 (−0.05, 2.67)	0.077
ESM ratio (cm^3^/cm^3^)		0.120		0.715
COPD vs PRISm				
COPD vs Normal				
Normal vs PRISm				
PMD_major_ (HU)		< 0.001		< 0.001
COPD vs PRISm	−3.61 (−7.21, −1.82)	< 0.001	−4.56 (−6.39, −2.73)	< 0.001
COPD vs Normal	−5.02 (−8.98, −2.20)	< 0.001	−4.58 (−6.42, −2.74)	< 0.001
Normal vs PRISm	1.41 (−1.27, 3.36)	0.176	0.02 (−1.73, 1.76)	0.536
PMD_minor_ (HU)		< 0.001		< 0.001
COPD vs PRISm	−2.44 (−6.69, −1.24)	< 0.001	−3.21 (−4.95, −1.46)	< 0.001
COPD vs Normal	−8.47 (−12.74, −6.15)	< 0.001	−5.72 (−7.64, −3.81)	< 0.001
Normal vs PRISm	6.03 (3.37, 7.73)	< 0.001	2.52 (0.68, 4.35)	< 0.01
ESMD (HU)		< 0.001		< 0.001
COPD vs PRISm	−5.99 (−8.74, −1.71)	< 0.001	−5.18 (−7.10, −2.11)	< 0.001
COPD vs Normal	−11.91 (−15.05, −7.75)	< 0.001	−6.71 (−9.21, −2.96)	< 0.001
Normal vs PRISm	5.93 (3.34, 8.25)	< 0.001	1.53 (−0.60, 3.50)	< 0.05

*PMVI*_*major*_ pectoralis major volume index, *PMVI*_*minor*_ pectoralis minor volume index, *ESM ratio* erector spinae muscle volume/the corresponding vertebral bone volume, *PMD*_*major*_ pectoralis major density, *PMD*_*minor*_ pectoralis minor density, *ESMD* erector spinae muscle density

**Table 4:** Comparison of 3D muscle parameters in female stratified by BMI

Variable	Female, BMI ≥ 25		Female, BMI < 25	
	Difference (95% CI)	Adjusted *p*-value	Difference (95% CI)	Adjusted *p*-value
PMVI_major_ (cm^3^/m^2^)		< 0.001		< 0.001
COPD vs PRISm	−4.41 (−8.37, 2.47)	0.394	−3.29 (−8.12, 1.53)	0.913
COPD vs Normal	−11.51 (−15.87, −5.17)	< 0.001	−7.55 (−10.99, −4.11)	< 0.01
Normal vs PRISm	7.10 (4.65, 11.48)	< 0.001	4.26 (−0.18, 8.69)	< 0.01
PMVI_minor_ (cm^3^/m^2^)		0.329		0.098
COPD vs PRISm				
COPD vs Normal				
Normal vs PRISm				
ESM ratio (cm^3^/cm^3^)		0.525		0.746
COPD vs PRISm				
COPD vs Normal				
Normal vs PRISm				
PMD_major_ (HU)		< 0.001		< 0.001
COPD vs PRISm	−8.88 (−11.54, −4.58)	< 0.01	−11.22 (−14.39, −8.19)	< 0.001
COPD vs Normal	−13.23 (−16.10, −9.08)	< 0.001	−11.88 (−13.65, −8.73)	< 0.001
Normal vs PRISm	4.35 (0.97, 7.58)	< 0.01	0.66 (−3.03, 2.69)	0.891
PMD_minor_ (HU)		< 0.05		< 0.001
COPD vs PRISm	−4.60 (−9.04, −0.17)	< 0.05	−9.37 (−12.40, −6.35)	< 0.001
COPD vs Normal	−5.62 (−10.00, −1.23)	< 0.05	−9.83 (−12.65, −7.00)	< 0.001
Normal vs PRISm	1.02 (−1.49, 3.52)	0.49	0.45 (−1.52, 2.43)	0.91
ESMD (HU)		< 0.001		< 0.001
COPD vs PRISm	−9.33 (−11.74, 0.12)	< 0.05	−11.30 (−16.62, −8.01)	< 0.001
COPD vs Normal	−13.92 (−17.33, −4.36)	< 0.001	−14.25 (−18.66, −11.65)	< 0.001
Normal vs PRISm	4.59 (1.33, 9.94)	< 0.01	2.95 (0.05, 5.48)	< 0.05

*PMVI*_*major*_ pectoralis major volume index, *PMVI*_*minor*_ pectoralis minor volume index, *ESM ratio* erector spinae muscle volume/the corresponding vertebral bone volume, *PMD*_*major*_ pectoralis major density, *PMD*_*minor*_ pectoralis minor density, *ESMD* erector spinae muscle density

Additionally, compared to the PRISm group, the normal group (male/female) showed higher PMVI_major_ (all *p* < 0.01) and ESM density (all *p* < 0.05). Some subgroups indicated that PM density in the normal group was higher than in the PRISm group.

#### Comparison of 3D muscle parameters across normal, PRISm, and COPD subjects stratified by age

In age-stratified analysis (Tables S3 and S4), the overall trend of intergroup comparisons was generally in line with the trend of BMI stratification.

However, male COPD subjects (age ≥ 65 years) had lower PMVI than the PRISm group (*p* < 0.05). Furthermore, only younger subjects in the normal group (age < 65 years) (male/female) had higher PMVI_major_ than in the PRISm group (both *p* < 0.001).

### Performance comparison of 3D and 2D models, and model explanation

**Fig. 5:**
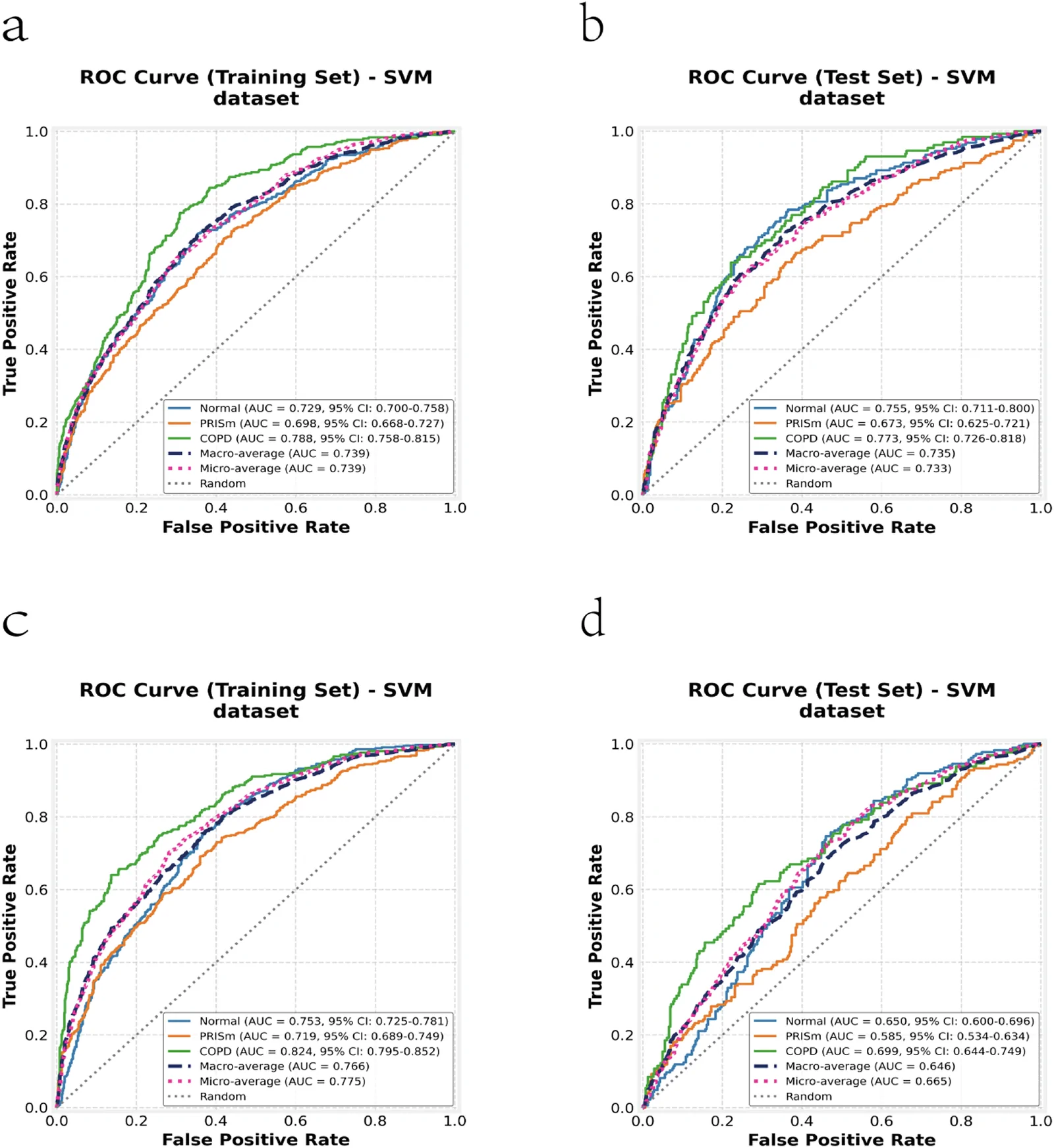
ROC curves of 3D and 2D SVM models for distinguishing normal, PRISm, and COPD patients using muscle parameters. **a** 3D training set, **b** 3D test set, **c** 2D training set, **d** 2D test set

In the test set, SVM-based classification analysis (Fig. 5) showed superior discrimination of the 3D muscle parameter model over the 2D model in discriminating normal, PRISm, and COPD groups. Specifically (Table 5), the macro-average AUC of the 3D model reached 0.735, significantly higher than 0.646 for the 2D model (*p* < 0.001), and the micro-average AUC for the 3D model was also significantly higher (0.733 vs 0.665, *p* < 0.001). Regarding classification performance for each group, the 3D model yielded significantly higher AUCs than the 2D model for the normal (0.755 vs 0.650), PRISm (0.673 vs 0.585), and COPD (0.773 vs 0.699) groups (all *p* < 0.05). Subgroup analysis by gender showed that the 3D SVM model maintained discriminative performance in both genders (Fig. S1). In the test set, macro-average AUCs were 0.705 (male) and 0.739 (female), with micro-average AUCs of 0.722 and 0.786, respectively.

**Table 5:** Comparison of classification performance for SVM models based on 3D and 2D muscle parameters in the testing cohort

Classification task	Parameter type	AUC	AUC [95% CI]	AUC difference	*p*-value (3D vs 2D)
COPD vs Rest	3D	0.773	0.726–0.818	0.074	< 0.05
	2D	0.699	0.644–0.749		
PRISm vs Rest	3D	0.673	0.625–0.721	0.088	< 0.05
	2D	0.585	0.534–0.634		
Normal vs Rest	3D	0.755	0.711–0.800	0.105	< 0.001
	2D	0.650	0.600–0.696		
Macro-average AUC	3D	0.735	_	0.089	< 0.001
	2D	0.646	_		
Micro-average AUC	3D	0.733	_	0.068	< 0.001
	2D	0.665	_		

*AUC* area under the receiver operating characteristic curve, *CI* confidence interval

**Fig. 6:**
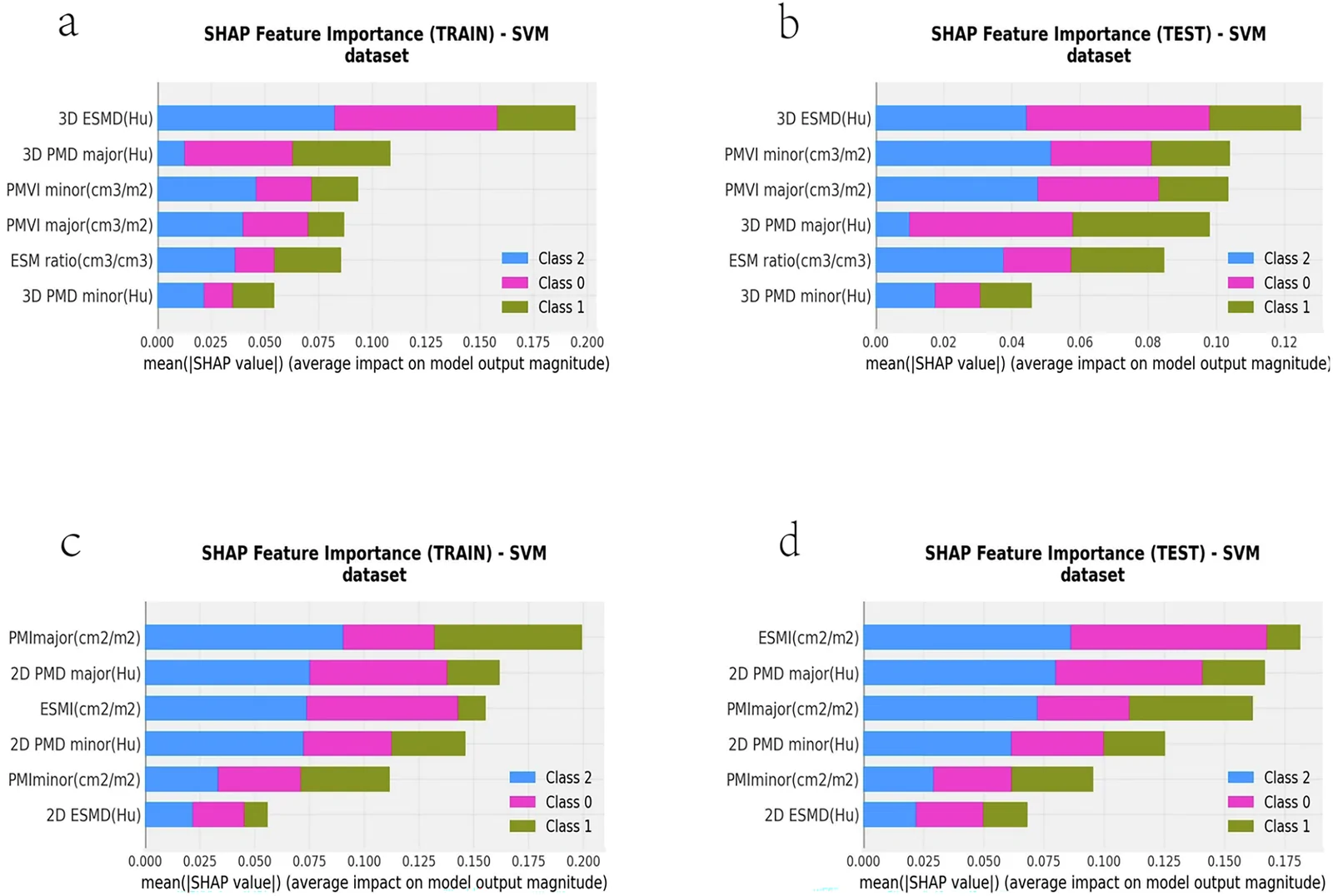
SHAP bar plots illustrate the importance of muscle parameters in the SVM models. **a** 3D training set, **b** 3D test set, **c** 2D training set, **d** 2D test set

The analysis of muscle parameter importance (3D/2D) based on SHAP values indicated differences in their distributions between the training set and test set. However, 3D ESMD showed relatively high importance among muscle parameters in both the training and test set (Fig. 6).

## Discussion

Sarcopenia significantly impairs pulmonary function and quality of life in COPD patients, necessitating early identification and management [31]. Muscle status in PRISm also warrants focused research. In this multicenter cohort, we quantified muscle parameters using both 3D and 2D segmentation. In the test set, the 3D SVM model demonstrated higher macro- and micro-average AUCs compared with the 2D model, with comparable performance observed in gender-stratified and overall analyses. SHAP analysis showed relatively high feature importance for 3D ESMD. Weak correlations between muscle parameters and pulmonary function (Table S2) were expected, as muscle changes in COPD and PRISm are mainly driven by systemic factors (inflammation, physical inactivity, comorbidities) beyond airflow limitation alone [5].

Recent TotalSegmentator studies reported Dice scores of 0.832–0.943 (Table S5). Although 2D segmentation is a powerful and convenient tool, and previous studies have primarily relied on this approach to analyze muscle parameters in COPD patients [15, 32, 33], muscle heterogeneity limits its ability to accurately assess overall muscle volume, as single-slice measurements show relatively large errors and residuals [34–36]. Additionally, 3D segmentation has gained popularity for its prognostic value. For example, Yi et al [27] developed a fully automated AI algorithm to extract volumetric features of six body components from cardiac CT for mortality prediction, identifying skeletal muscle volume index as a protective factor (adjusted HR 0.56). Hathaway et al [37] used TotalSegmentator for automated 3D vertebral bone mineral density (BMD) quantification, showing that 3D BMD combined with the Fracture Risk Assessment Tool with no BMD (FRAXnb) improved fracture prediction (AUC 0.82) over FRAXnb alone (AUC 0.66) and 2D measurements (axial AUC 0.74, sagittal AUC 0.73). Our study also quantified PM and ESM volumes using the TotalSegmentator, and reduced variability from incomplete ESM coverage using vertebral volume–based normalization. The findings provided an alternative imaging-based analytical approach for evaluating individuals with impaired lung function.

Additionally, 3D and subgroup analyses showed muscle alterations from normal to PRISm to COPD for some parameters, with more consistent changes in density than volume parameters. Males showed more pronounced differences in muscle volume across groups, whereas females exhibited mainly a decline in muscle density, consistent with Anderson et al [38]. The subgroup analysis revealed that COPD patients exhibited lower muscle density compared to PRISm. Moreover, when stratified by BMI, no significant differences in muscle volume were observed between groups. This suggests the decline in muscle density may occur earlier in the disease process, which was further supported by previous studies indicating a stronger association between muscle density and pulmonary function [39], as well as the relatively low prevalence of muscle atrophy in patients with COPD [40]. In gender-stratified 3D SVM analysis, the model performed slightly better in females, possibly due to greater sensitivity to muscle density changes. However, given the small female sample size and gender imbalance, this finding may reflect sampling variability rather than a true gender-specific effect.

Furthermore, we found that regardless of the stratification method used, the 3D ESMD in the PRISm group was lower than that in the normal group, which was consistent with the results obtained from SHAP analysis. Tanabe et al [41] also mentioned in their study that ESM, as an anti-gravity muscle, its CSA can serve as a simple and non-invasive imaging biomarker for assessing systemic muscle consumption. Through our 3D analysis visualization, this phenomenon is illustrated, providing additional insight.

This study has several limitations. First, as a retrospective study, the unequal distribution of normal, PRISm, and COPD subjects across centers may have introduced selection bias, and the study lacked data on key confounders (physical activity, nutrition, lipid metabolism, and hormones) important for muscle assessment [42, 43]. In addition, kernel SHAP interpretation for SVM models may be influenced by sample size and computational settings. Second, only 77 female COPD patients were included because COPD is more prevalent in males [1], and there was a significant gender distribution difference between the PRISm group and the normal control group, causing gender imbalance. Third, ESM analysis was restricted to the chest CT field without full spinal coverage, but we used vertebral bone volume as an internal reference to minimize inter-individual variability. Finally, we analyzed only muscle density without evaluating subcutaneous or visceral fat, both of which have established prognostic value [10, 44]. Given that PRISm may be a heterogeneous state that could progress to COPD, future studies incorporating comprehensive clinical variables, whole-spine imaging, and full body composition analysis are needed for clinically applicable predictive models.

In conclusion, the chest CT–based 3D muscle parameters model showed better discrimination of normal, PRISm, and COPD subjects than the 2D model. 3D ESMD showed relatively high SHAP importance. These findings support the potential value of 3D muscle quantification in individuals with impaired pulmonary function.

## Supplementary information


Electronic Supplementary Material


## Data Availability

The datasets generated or analyzed during this study are available from the corresponding author upon reasonable request.

## Abbreviations

AUC: Area under the curve
COPD: Chronic obstructive pulmonary disease
ESM: Erector spinae muscle
FEV_1_: Forced expiratory volume in 1 s
FEV_1_% predicted: Forced expiratory volume in 1 s expressed as percent predicted
FVC: Forced vital capacity
GOLD: Global Initiative for Chronic Obstructive Lung Disease
PM: Pectoralis muscle
PRISm: Preserved ratio impaired spirometry
SVM: Support vector machine

## References

[1] Boers E, Barrett M, Su JG et al (2023) Global burden of chronic obstructive pulmonary disease through 2050. JAMA Netw Open 6:e2346598. 10.1001/jamanetworkopen.2023.46598 38060225 PMC10704283

[2] Soriano JB, Kendrick PJ, Paulson KR et al (2020) Prevalence and attributable health burden of chronic respiratory diseases, 1990–2017: a systematic analysis for the Global Burden of Disease Study 2017. Lancet Respir Med 8:585–596. 10.1016/s2213-2600(20)30105-3 32526187 PMC7284317

[3] Wang C, Xu J, Yang L et al (2018) Prevalence and risk factors of chronic obstructive pulmonary disease in China (the China Pulmonary Health [CPH] study): a national cross-sectional study. Lancet 391:1706–1717. 10.1016/s0140-6736(18)30841-9 29650248

[4] Agustí A, Celli BR, Criner GJ et al (2023) Global Initiative for Chronic Obstructive Lung Disease 2023 report: GOLD executive summary. Am J Respir Crit Care Med 207:819–837. 10.1164/rccm.202301-0106pp 36856433 PMC10111975

[5] Celli BR, Casanova C, Plata VP (2004) The body-mass index, airflow obstruction, dyspnea, and exercise capacity index in chronic obstructive pulmonary disease. N Engl J Med 350:1005–1012 10.1056/NEJMoa021322 14999112

[6] Jones PW (2001) Health status measurement in chronic obstructive pulmonary disease. Thorax 56:880–887. 10.1136/thorax.56.11.880 11641515 PMC1745959

[7] Greening NJ, Harvey-Dunstan TC, Chaplin EJ et al (2015) Bedside assessment of quadriceps muscle by ultrasound after admission for acute exacerbations of chronic respiratory disease. Am J Respir Crit Care Med 192:810–816. 10.1164/rccm.201503-0535oc 26068143 PMC4613897

[8] Swallow EB, Reyes D, Hopkinson NS et al (2007) Quadriceps strength predicts mortality in patients with moderate to severe chronic obstructive pulmonary disease. Thorax 62:115–120. 10.1136/thx.2006.062026 17090575 PMC2111256

[9] Martinez CH, Diaz AA, Meldrum CA et al (2017) Handgrip strength in chronic obstructive pulmonary disease. Associations with acute exacerbations and body composition. Ann Am Thorac Soc 14:1638–1645. 10.1513/annalsats.201610-821oc 29090990 PMC5711268

[10] Pishgar F, Shabani M, Silva TQAC et al (2021) Quantitative analysis of adipose depots by using chest CT and associations with all-cause mortality in chronic obstructive pulmonary disease: longitudinal analysis from MESArthritis ancillary study. Radiology 299:703–711. 10.1148/radiol.2021203959 33825508 PMC8165946

[11] Wilson AC, Bon JM, Mason S et al (2022) Increased chest CT derived bone and muscle measures capture markers of improved morbidity and mortality in COPD. Respir Res 23:311. 10.1186/s12931-022-02237-w 36376854 PMC9664607

[12] Li C, Lian X, He J et al (2024) Association of computed tomography-derived pectoralis muscle area and density with disease severity and respiratory symptoms in patients with chronic obstructive pulmonary disease: a case-control study. Respir Med 233:107783. 10.1016/j.rmed.2024.107783 39209127

[13] Bak SH, Kwon SO, Han S-S, Kim WJ (2019) Computed tomography-derived area and density of pectoralis muscle associated disease severity and longitudinal changes in chronic obstructive pulmonary disease: a case control study. Respir Res 20:226. 10.1186/s12931-019-1191-y 31638996 PMC6805427

[14] Tanimura K, Sato S, Fuseya Y et al (2016) Quantitative assessment of erector spinae muscles in patients with chronic obstructive pulmonary disease. Novel chest computed tomography–derived index for prognosis. Ann Am Thorac Soc 13:334–341. 10.1513/AnnalsATS.201507-446OC 26700501

[15] Nakagawara K, Shiraishi Y, Chubachi S et al (2024) Integrated assessment of computed tomography density in pectoralis and erector spinae muscles as a prognostic biomarker for coronavirus disease 2019. Clin Nutr 43:815–824. 10.1016/j.clnu.2024.02.004 38350289

[16] Wong J, Huang V, Wells D et al (2021) Implementation of deep learning-based auto-segmentation for radiotherapy planning structures: a workflow study at two cancer centers. Radiat Oncol 16:101. 10.1186/s13014-021-01831-4 34103062 PMC8186196

[17] Li H, Zhang H, Johnson H et al (2021) Longitudinal subcortical segmentation with deep learning. Proc SPIE Int Soc Opt Eng 11596:115960D 10.1117/12.2582340 PMC8643360 34873358

[18] Lenchik L, Heacock L, Weaver AA et al (2019) Automated segmentation of tissues using CT and MRI: a systematic review. Acad Radiol 26:1695–1706. 10.1016/j.acra.2019.07.006 31405724 PMC6878163

[19] Wan ES (2022) The clinical spectrum of PRISm. Am J Respir Crit Care Med 206:524–525. 10.1164/rccm.202205-0965ed 35612910 PMC9716910

[20] Wan ES, Fortis S, Regan EA et al (2018) Longitudinal phenotypes and mortality in preserved ratio impaired spirometry in the COPDGene study. Am J Respir Crit Care Med 198:1397–1405. 10.1164/rccm.201804-0663OC 29874098 PMC6290948

[21] Wan ES, Balte P, Schwartz JE et al (2021) Association between preserved ratio impaired spirometry and clinical outcomes in US adults. JAMA 326:2287. 10.1001/jama.2021.20939 34905031 PMC8672237

[22] Perez-Padilla R, Montes De Oca M, Thirion-Romero I et al (2023) Trajectories of spirometric patterns, obstructive and PRISm, in a population-based cohort in Latin America. COPD 18:1277–1285. 10.2147/copd.s406208 PMC10290847 37366430

[23] Global Initiative for Chronic Obstructive Lung Disease. Global Strategy for the Diagnosis, Management, and Prevention of Chronic Obstructive Pulmonary Disease: 2025 Report. 2024. Available from: https://goldcopd.org/2025-gold-report/

[24] Wasserthal J, Breit H-C, Meyer MT et al (2023) TotalSegmentator: robust segmentation of 104 anatomic structures in CT images. Radiol Artif Intell 5:e230024. 10.1148/ryai.230024 37795137 PMC10546353

[25] Borys K, Lodde G, Livingstone E et al (2025) Fully volumetric body composition analysis for prognostic overall survival stratification in melanoma patients. J Transl Med 23:532. 10.1186/s12967-025-06507-1 40355935 PMC12067685

[26] Tassani S, Öhman C, Baruffaldi F et al (2011) Volume to density relation in adult human bone tissue. J Biomech 44:103–108. 10.1016/j.jbiomech.2010.08.032 20850118

[27] Yi J, Marcinkiewicz AM, Shanbhag A et al (2025) AI-based volumetric six-tissue body composition quantification from CT cardiac attenuation scans for mortality prediction: a multicentre study. Lancet Digit Health 7:100862. 10.1016/j.landig.2025.02.002 40382274 PMC12126277

[28] Maetani T, Tanabe N, Shiraishi Y et al (2023) Centrilobular emphysema is associated with pectoralis muscle reduction in current smokers without airflow limitation. Respiration 102:194–202. 10.1159/000529031 36689922 PMC10064397

[29] Teigen LM, John R, Kuchnia AJ et al (2017) Preoperative pectoralis muscle quantity and attenuation by computed tomography are novel and powerful predictors of mortality after left ventricular assist device implantation. Circ Heart Fail 10:e004069. 10.1161/CIRCHEARTFAILURE.117.004069 28912261

[30] Della Peruta C, Lozanoska-Ochser B, Renzini A et al (2023) Sex differences in inflammation and muscle wasting in aging and disease. Int J Mol Sci 24:4651. 10.3390/ijms24054651 36902081 PMC10003083

[31] Sepúlveda-Loyola W, Osadnik C, Phu S et al (2020) Diagnosis, prevalence, and clinical impact of sarcopenia in COPD: a systematic review and meta-analysis. J Cachexia Sarcopenia Muscle 11:1164–1176. 10.1002/jcsm.12600 32862514 PMC7567149

[32] McDonald M-LN, Diaz AA, Ross JC et al (2014) Quantitative computed tomography measures of pectoralis muscle area and disease severity in chronic obstructive pulmonary disease. A cross-sectional study. Ann Am Thorac Soc 11:326–334. 10.1513/AnnalsATS.201307-229OC 24558953 PMC4028743

[33] Zhou K, Wu F, Zhao N et al (2025) Association of pectoralis muscle area on computed tomography with airflow limitation severity and respiratory outcomes in COPD: a population-based prospective cohort study. Pulmonology 31:2416782. 10.1016/j.pulmoe.2023.02.004 36907812

[34] Diaz AA, Zhou L, Young TP et al (2014) Chest CT measures of muscle and adipose tissue in COPD. Acad Radiol 21:1255–1261. 10.1016/j.acra.2014.05.013 25088837 PMC4389286

[35] MacDonald AJ, Greig CA, Baracos V (2011) The advantages and limitations of cross-sectional body composition analysis. Curr Opin Support Palliat Care 5:342–349. 10.1097/SPC.0b013e32834c49eb 21986910

[36] Ottenheijm CAC, Heunks LMA, Dekhuijzen PNR (2007) Diaphragm muscle fiber dysfunction in chronic obstructive pulmonary disease: toward a pathophysiological concept. Am J Respir Crit Care Med 175:1233–1240. 10.1164/rccm.200701-020PP 17413128

[37] Hathaway QA, Kasaeian A, Pan T et al (2025) A deep learning model for three-dimensional determination of whole thoracic vertebral bone mineral density from noncontrast chest CT: the multi-ethnic study of atherosclerosis. Radiology 314:e242133. 10.1148/radiol.242133 40067103 PMC11950887

[38] Anderson LJ, Liu H, Garcia JM (2017) Sex differences in muscle wasting. In: Mauvais-Jarvis F (ed) Sex and gender factors affecting metabolic homeostasis, diabetes and obesity. Springer, Cham, pp 153–197

[39] Jung YJ, Lee MJ, Kim EH et al (2025) Association of lung function with visceral adiposity and skeletal muscle mass considering myosteatosis. Chest 167:1714–1726. 10.1016/j.chest.2024.12.018 39788316

[40] Mason SE, Moreta-Martinez R, Labaki WW et al (2021) Respiratory exacerbations are associated with muscle loss in current and former smokers. Thorax 76:554–560. 10.1136/thoraxjnl-2020-215999 33574123 PMC8222105

[41] Tanabe N, Sato S, Tanimura K et al (2021) Associations of CT evaluations of antigravity muscles, emphysema and airway disease with longitudinal outcomes in patients with COPD. Thorax 76:295–297. 10.1136/thoraxjnl-2020-215085 32868293

[42] Tuñón-Suárez M, Reyes-Ponce A, Godoy-Órdenes R et al (2021) Exercise training to decrease ectopic intermuscular adipose tissue in individuals with chronic diseases: a systematic review and meta-analysis. Phys Ther 101:pzab162. 10.1093/ptj/pzab162 34174085

[43] Hua N, Qin C, Wu F et al (2024) High-density lipoprotein cholesterol level and risk of muscle strength decline and sarcopenia in older adults. Clin Nutr 43:2289–2295. 10.1016/j.clnu.2024.08.017 39217844

[44] Liao Z, Yuan G, Kangwen He et al (2024) Body composition as a potential imaging biomarker for predicting the progression risk of chronic kidney disease. Insights Imaging 15:247. 10.1186/s13244-024-01826-1 39400628 PMC11473763

