# Supplementary material for: Automated 3D chest CT muscle segmentation–derived model development and explanation in discrimination of normal, preserved ratio impaired spirometry, and COPD: a multicenter study
Source: Insights Imaging. 2026 Aug 27;17:214. doi: 10.1186/s13244-026-02384-4 (PMC13522301; doi:10.1186/s13244-026-02384-4)
Supplement: Supplementary file 1 [file 13244_2026_2384_MOESM1_ESM.pdf]

Automated 3D chest CT muscle segmentation–derived model development and explanation in discrimination of normal, preserved ratio impaired spirometry, and COPD: a multicenter study

Electronic Supplementary Material

Automated 3D Chest CT Muscle Segmentation–Derived Model Development and Explanation in Discrimination of Normal, PRISm and COPD: a Multicenter Study

List:

Table S1: CT protocols of the four centers

Table S2: Partial correlation analysis between pulmonary function and 3D muscle parameters

TableS3: Comparison of 3D muscle Parameters in male stratified by age

TableS4: Comparison of 3D muscle Parameters in female stratified by age

Table S5 Muscle segmentation performance of TotalSegmentator in different studies

Figure S1: ROC curves of the 3D SVM model for discriminating normal, PRISm, and COPD patients by gender **(a)** male training set, **(b)** male test set, **(c)** female training set, **(d)** female test set

Table S1 CT protocols of the four centers

Scanning equipment	Light speed 64VCT	Philips brilliance iCT	Philips ingenuity CT	GE Optima CT
	(Center1)<br>(Center4)	(Center1)<br>(Center4)	(Center1)<br>(Center4)	(Center2)<br>(Center3)
Tube voltage (kV)	120<br>120	120<br>120	120<br>120	120<br>120
Tube current (mAs)	30<br>30	AEC<br>AEC	AEC<br>AEC	200<br>ATCM
Pitch	0.8<br>0.8	0.8<br>0.8	1.0<br>1.0	1.2<br>1.2
Collimation (mm)	0.6 × 64<br>0.6 × 64	0.6 × 128<br>0.6 × 128	0.6 × 128<br>0.6 × 128	0.6 × 64<br>0.6 × 64
Slice thickness (mm)	5<br>5	5<br>5	5<br>5	1<br>1

AEC, dose modulation with automatic exposure Control; GE, General Electric; ATCM, Automatic Tube Current Modulation

Table S2 Partial correlation analysis between pulmonary function and 3D muscle parameters

Muscle parameters	FEV1% predicted			FEV1/FVC%		
	r	CI 95%	p	r	CI 95%	p
PMVI $_{major}$ (cm^3/m^2)	0.102	0.05-0.16	<0.001	0.136	0.08-0.19	<0.001
PMVI $_{minor}$ (cm^3/m^2)	0.015	-0.04-0.07	0.607	0.071	0.02-0.13	<0.05
ESM ratio(cm^3/cm^3)	-0.017	-0.07-0.04	0.547	0.026	-0.03-0.08	0.360
3D PMD $_{major}$ (Hu)	0.090	0.03-0.14	<0.01	0.291	0.24-0.34	<0.001
3D PMD $_{minor}$ (Hu)	0.051	0.00-0.11	0.073	0.214	0.16-0.27	<0.001
3D ESMD (Hu)	0.138	0.08-0.19	<0.001	0.295	0.24-0.34	<0.001

FEV1% predicted, forced expiratory volume in 1 s expressed as percent predicted; FEV1/FVC%, forced expiratory volume during the first second of forced breath [FEV1] / forced vital capacity [FVC]; PMVI $_{major}$, pectoralis major volume index; PMVI $_{minor}$, pectoralis minor volume index; ESM ratio, erector spinae muscle volume /the corresponding vertebral bone volume; PMD $_{major}$, pectoralis major density; PMD $_{minor}$, pectoralis minor density; ESMD, erector spinae muscle density

TableS3 Comparison of 3D muscle Parameters in male stratified by age

Variables	Male，Age≥65		Male，Age<65	
	Difference (95% CI)	Adjusted p value	Difference (95% CI)	Adjusted p value
PMVI$_{major}$ (cm^3/m^2)		<0.05		<0.001
COPD VS PRISm	-4.61（-8.82, -0.41）	<0.05	-2.02（-7.49, 3.45）	0.768
COPD VS Normal	-4.95（-9.92, 0.02）	<0.05	-10.73（-15.67, -5.78）	<0.001
Normal VS PRISm	0.34（-4.63, 5.31）	0.741	8.71（3.92, 13.50）	<0.001
PMVI$_{minor}$ (cm^3/m^2)		<0.01		<0.05
COPD VS PRISm	-1.84 (-3.20, -0.88)	<0.01	-0.69 (-2.37, 1.29)	0.778
COPD VS Normal	-1.99 (-2.96, -0.89)	<0.01	-2.46 (-3.55, -0.53)	<0.05
Normal VS PRISm	0.15 (-1.21, 1.11)	0.907	1.76 (0.04, 3.07)	<0.05
ESM ratio(cm^3/cm^3)		0.214		0.113
COPD VS PRISm				
COPD VS Normal				
Normal VS PRISm				
PMD$_{major}$ (Hu)		<0.001		<0.01
COPD VS PRISm	-2.35(-3.96, -1.04)	<0.001	-3.36（-5.82, -0.90）	<0.01
COPD VS Normal	-2.83(-4.41, -1.06)	<0.001	-3.68（-6.08, -1.28）	<0.01
Normal VS PRISm	0.49 (-1.50, 2.04)	0.869	0.32（-1.46, 2.10）	0.812
PMD$_{minor}$ (Hu)		<0.05		<0.05
COPD VS PRISm	-0.31(-1.88, 1.12)	<0.05	-3.14(-6.45, -1.32)	<0.05
COPD VS Normal	-1.24(-3.19, 0.19)	<0.05	-3.51(-6.50, -0.97)	<0.05
Normal VS PRISm	0.93(-0.50, 3.08)	0.278	0.38 (-2.42, 2.10)	0.776
ESMD (Hu)		<0.001		<0.01
COPD VS PRISm	-4.08 (-6.31, -2.44)	<0.001	-0.80（-3.21, 1.61）	0.675
COPD VS Normal	-6.27 (-9.34, -4.50)	<0.001	-3.29（-5.70, -0.89）	<0.05
Normal VS PRISm	2.19 (0.37, 4.55)	<0.05	2.49（0.78, 4.21）	<0.05

PMVI $_{major}$, pectoralis major volume index; PMVI $_{minor}$, pectoralis minor volume index; ESM ratio, erector spinae muscle volume /the corresponding vertebral bone volume; PMD $_{major}$, pectoralis major density; PMD $_{minor}$, pectoralis minor density; ESMD, erector spinae muscle density

TableS4 Comparison of 3D muscle Parameters in female stratified by age

Variables	Female，Age≥65		Female，Age<65	
	Difference (95% CI)	Adjusted p value	Difference (95% CI)	Adjusted p value
PMVI $_{major}$ (cm^3/m^2)		<0.01		<0.001
COPD VS PRISm	-2.60(-8.58, 0.83)	0.211	-0.83 (-6.08, 4.43)	0.949
COPD VS Normal	-4.27(-9.71, -1.88)	<0.05	-8.92 (-13.96, -3.87)	<0.05
Normal VS PRISm	1.67(-1.87, 5.83)	=0.098	8.09 (4.65, 11.54)	<0.001
PMVI $_{minor}$ (cm^3/m^2)		0.591		0.171
COPD VS PRISm				
COPD VS Normal				
Normal VS PRISm				
ESM ratio(cm^3/cm^3)		0.325		0.148
COPD VS PRISm				
COPD VS Normal				
Normal VS PRISm				
PMD $_{major}$(Hu)		<0.001		<0.001
COPD VS PRISm	-8.33 (-12.54, -4.40)	<0.001	-8.23 (-13.86, -3.61)	<0.001
COPD VS Normal	-9.06 (-12.42, -6.31)	<0.001	-11.47 (-16.03, -7.50)	<0.001
Normal VS PRISm	0.74 (-2.87, 4.27)	0.439	3.24 (-0.12, 6.08)	<0.001
PMD $_{minor}$(Hu)		<0.01		<0.001
COPD VS PRISm	-4.17 (-7.61, -0.74)	<0.01	-7.03 (-12.57, -1.87)	<0.01
COPD VS Normal	-4.39 (-7.62, -1.16)	<0.01	-10.74 (-15.02, -6.30)	<0.001
Normal VS PRISm	0.21 (-2.16, 2.59)	0.652	3.71 (-0.81, 6.25)	0.093
ESMD (Hu)		<0.001		<0.001
COPD VS PRISm	-4.69 (-9.33, 0.89)	<0.05	-12.32 (-18.52, -5.72)	<0.001
COPD VS Normal	-8.78 (-12.82, -3.85)	<0.001	-16.94 (-21.91, -10.49)	<0.001
Normal VS PRISm	4.09 (0.43, 7.09)	<0.05	4.61 (2.02, 6.79)	<0.001

PMVI $_{major}$, pectoralis major volume index; PMVI $_{minor}$, pectoralis minor volume index; ESM ratio, erector spinae muscle volume /the corresponding vertebral bone volume; PMD $_{major}$, pectoralis major density; PMD $_{minor}$, pectoralis minor density; ESMD, erector spinae muscle density

Table S5 Muscle segmentation performance of TotalSegmentator in different studies

Study	Muscle type	Segmentation Approach	Dice Score
Wasserthal et al. (2023）	Autochthon ,Iliopsoas Gluteus, et al	TotalSegmentator (nnU-Net-based)	0.943(0.938,0.947) (high-res) 0.840(0.836,0.844) (low-res)
Hou et al. (2024）	CT field-of-view skeletal muscle	TotalSegmentator	0.832±0.046 (0.805, 0.864)

CT field-of-view skeletal muscle: Total volume of all skeletal muscles within the CT field of view, segmented as a single semantic class. Scan coverage varied across patients (e.g., thorax, abdomen, pelvis) due to dataset (SAROS) heterogeneity.

Supplementary reference:

[1] HOU B, MATHAI T S, LIU J, et al. Enhanced muscle and fat segmentation for CT-based body composition analysis: a comparative study[J/OL]. International Journal of Computer Assisted Radiology and Surgery, 2024, 19(8): 1589-1596. DOI:10.1007/s11548-024-03167-2.

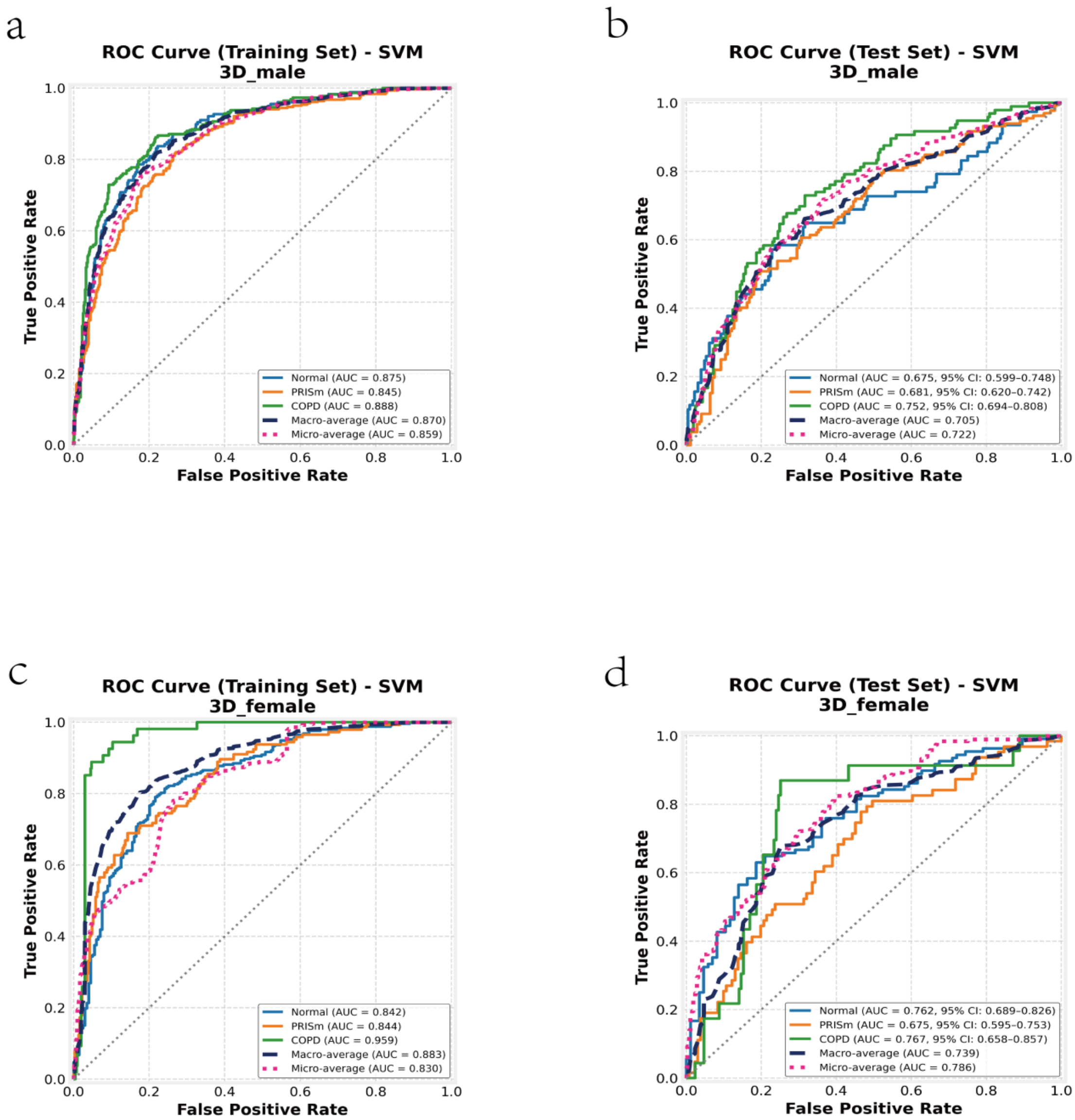

Figure S1:ROC curves of the 3D SVM model for discriminating normal, PRISm, and COPD patients by gender **(a)** male training set, **(b)** male test set, **(c)** female training set, **(d)** female test set
